# Scientific Items

**Published:** 1882-05-20

**Authors:** 


### SCIENTIFIC ITEMS.

**The Hygienic Value of the Electric Light.**—The French Scientific Journal La Nature summarizes a communication from Dr. Javal, who believes that the electric light is absolutely without danger to the sight, in consequence of the amount of division which can now be obtained in it. L'Union Midicale also reminds its readers that similar researches of great interest from a scholastic point of view were published in that journal in May and July, 1881; including the researches of Dr. Cohn of Breslau, who found that the electric light increases sixfold, as compared with daylight, the perception of yellow, and doubles the perception of green and blue. The observations of Dr. Blasius and Dr. Hopper in a discussion which took place at a meeting of the Brunswick Society of Natural Sciences, are also noteworthy. These scientists have shown that illumination by the electric light deserved preference over all other methods in use, for the following reasons: 1. It does not pollute the air with deleterious gases or other unhealthy products. 2. It induces a greater visual unity than with daylight or gaslight. The conclusion adopted by the meeting was, that "the hygienic qualities of the electric light have not hitherto been appraised at their real value."—*British Medical Journal.*

**A Naval Experiment With the Electric Light.**—The Providence Journal gives an account of a trial of the electric light as used to detect the movements of vessels at night, especially torpedo boats in time of war. The light is placed in a parabolic reflector, which is pivoted to turn in any desired direction and moved by a small electric engine in the horizontal plains of the motion.

The experiment was directed by Captain Selfridge, of the United States Navy, and with the United States steamship Nina and a small steam launch from the torpedo station of Newport, R. I. The launch was sent to the outer harbor, followed after some time by the Nina, fitted with a light on each side, to seek for her in the darkness. The launch was to play around and approach with muffled ores and hidden lights as near as possible to the Nina without being heard. The little craft was promptly detected at considerable distrance as soon as the light swept over her locality.—*Scientific News.*

**Fecundity of Elephants in Confinement.**—Barnum's baby elephant, born at Philadelphia, is growing rapidly, and is a vigorous creature in its third year. Its mother is again about seven months along in pregnancy, and it is therefore not unlikely that a second one will be born in this country. The sexes in Barnum's herd of elephants pair readily in confinement. We learn on good authority that an American resident in India of thirty years, never heard of an elephant being born there, and was astonished at learning of the birth of one in the United States.—*American Naturalist.*

**Cotton Fiber.**—A new staple of manufacture consists of the fiber of the stalks of the cotton plant. The stalk is disintegrated, and the fiber separated from the rest of the stalk, preserved and prepared according to the following method: First, separating the fiber from the stalk by passing through rollers, or by setting, then drying, then stretching or breaking, and then carding or hacking the same, thus producing a staple of the fiber alone. It is proposed to manufacture from this staple woven fabrics by spinning it, and twine, cordage and yarns, wadding, packing, calking and paper.—*Mechanical News.*

A LATE invention provides for extinguishing fires in the stoves of passenger cars, should they be by accident upset. For this purpose an elongated water-reservoir is suspended along the side of the car. Cords attached to the stove, and to valves which normally close outside in the reservoir, serve to operate said valves when the stove is upset, and allow water, through a perforated distributor, to flow into the fire.—*Ibid.*

THE process of whitening sugar was discovered in a curious way. A hen that had gone through a clay puddle meandered thence into a sugar-house. She left her tracks on a pile of sugar. It was noticed that wherever the tracks were the sugar was whitened. Experiments were instituted, and the result was that wet clay came to be used in refining sugar.—*Medical News.*

THE New York Fire Commissioners have experimented with asbestos as a material for fire-proof stage-curtains, and found it satisfactory. It will resist heat, without burning, long enough to allow any theater audience to leave before the fire could break out beyond the stage. No report has as yet been made, and the Commissioners demand as the final test that it shall be shown to their satisfaction that an asbestos curtain of the required size shall sustain its own weight. If this can be proved, legislative action will be asked to compel managers of theaters to adopt such curtains.—*Ibid.*

THE tunneling of the English channel is progessing so well that the first quarter of a mile is now completed. What looks especially encouraging is that the engineers are able to gradually increase the rate of boring which has attained the excellent average of 36 feet per day; and, in addition to this, the soil is now quite dry, there being a total absence of springs, the presence of which proved a source of much delay in the Abbot's Cliff heading. There are now about sixty men engaged on the works. They are employed in two night and day shifts, but it is proposed shortly to have an extra shift, making eight hours each, in order to expedite the work. No boring is done on Sunday, the men being chiefly employed on that day in lengthening the double line of metal in which the trollies carrying the debris travel. The boring has now advanced several yards under the sea in the direction of the Admiralty Pier at Dover.—*Ibid.*

